# Cell Adhesion and Long-Term Survival of Transplanted Mesenchymal Stem Cells: A Prerequisite for Cell Therapy

**DOI:** 10.1155/2015/632902

**Published:** 2015-02-02

**Authors:** Seahyoung Lee, Eunhyun Choi, Min-Ji Cha, Ki-Chul Hwang

**Affiliations:** ^1^Institute for Bio-Medical Convergence, College of Medicine, Catholic Kwandong University, Gangneung-si, Gangwon-do 210-701, Republic of Korea; ^2^Catholic Kwandong University International St. Mary's Hospital, Incheon Metropolitan City 404-834, Republic of Korea

## Abstract

The literature provides abundant evidence that mesenchymal stem cells (MSCs) are an attractive resource for therapeutics and have beneficial effects in regenerating injured tissues due to their self-renewal ability and broad differentiation potential. Although the therapeutic potential of MSCs has been proven in both preclinical and clinical studies, several questions have not yet been addressed. A major limitation to the use of MSCs in clinical applications is their poor viability at the site of injury due to the harsh microenvironment and to anoikis driven by the loss of cell adhesion. To improve the survival of the transplanted MSCs, strategies to regulate apoptotic signaling and enhance cell adhesion have been developed, such as pretreatment with cytokines, growth factors, and antiapoptotic molecules, genetic modifications, and hypoxic preconditioning. More appropriate animal models and a greater understanding of the therapeutic mechanisms of MSCs will be required for their successful clinical application. Nevertheless, the development of stem cell therapies using MSCs has the potential to treat degenerative diseases. This review discusses various approaches to improving MSC survival by inhibiting anoikis.

## 1. Introduction

Regenerative medicine is defined as the process of replacing or regenerating damaged cells, tissues, and/or organs to restore normal function [1], and one recently established stem cell-based therapy has the potential to treat many degenerative diseases and age-related diseases [2]. Generally, stem cells can be classified as embryonic stem cells (ESCs), adult stem cells (ASCs), which include mesenchymal stem cells (MSCs), hematopoietic stem cells (HSCs), and tissue/organ-specific stem/progenitor cells [3], and induced pluripotent stem cells (iPSCs), which can be produced by converting somatic cells into an ESC-like pluripotent state through the genetic modification of transcription factor expression [4, 5]. Although ESCs and iPSCs have useful properties, such as pluripotency, unlimited quantity, and wide biomedical applications in cell therapy, their clinical relevance has been impeded by ethical considerations, safety issues such as tumorigenicity and immunogenic response, low efficiency, and limited accessibility [3, 6, 7].

Unlike ESCs, ASCs have no ethical issues attached to their use [7, 8], and these cells are essential to maintaining homeostasis by renewing and/or regenerating damaged tissues under physiological and pathological conditions; therefore, the use of ASCs in stem cell therapy is an alternative strategy for clinical therapeutics [9]. Among the different types of ASCs, MSCs have attracted interest for researchers in the fields of stem cell therapy, because MSCs can be easily isolated from bone marrow, adipose tissue, synovium, periosteum, tooth, and placenta [10, 11], and expanded with high efficiency [10]. Additionally, MSCs not only have the capacity to differentiate into a variety of cell lineages under defined environmental conditions [12] but also exhibit immunosuppressive effects, which permits their successful transplantation to an allogeneic (a compatible donor) graft [13]. The study by Devine et al. demonstrated that allogeneic MSCs were not rejected and were associated with outcomes similar to those of autologous (self-derived) MSCs in nonhuman primates [14, 15]. Consequently, these characteristics make MSCs suitable for therapeutic use, and many preclinical studies of the therapeutic application of MSCs have demonstrated their beneficial effects [16, 17]. In addition, one hundred clinical trials using MSCs were ongoing in 2011 [18, 19]. These clinical studies represent a broad spectrum of MSC applications, including the treatment of diseases such as severe graft-versus-host disease (GVHD) [20], severe osteogenesis imperfecta [21], and metachromatic leukodystrophy (MLD) and Hurler syndrome (MPS-IH) [22, 23], as well as the treatment of chronically injured hearts [24].

Despite the impressive potential of the MSC-based therapy, several obstacles (e.g., the difficulty of maintaining self-renewal and poor survival due to apoptosis and/or necrosis at the administration site) have been encountered [25]. The primary limitation is the poor viability (low survival rates) of the transplanted MSCs by anoikis in injured tissues. Anoikis is a form of programmed cell death that occurs due to the loss of anchorage-dependent attachment to the extracellular matrix (ECM) [26, 27]. Because cell-cell adhesion through the ECM plays an important role in cell activities, proliferation, and survival [28], a low propensity to adhere to the host cells due to a loss of matrix anchorage may induce the death of the transplanted MSCs. Although several ongoing studies are focused on improving MSC survival, no potential solutions have been suggested to solve the underlying problem of weak adhesion.

In this review, we focus on the survival and adhesion of the transplanted MSCs. Cell adhesion is associated with cell survival; therefore, enhancing the adhesion and survival of the transplanted MSCs through the inhibition of anoikis should improve the success of MSC-based clinical applications.

## 2. Regeneration Mechanisms of Transplanted MSCs

This section provides a brief discussion of how transplanted MSCs exert their beneficial effects, before addressing the main subject of the review. The regenerative mechanisms of the transplanted MSCs in damaged tissues are not fully understood; however, some reports have suggested potential mechanisms including cell fusion, differentiation, and paracrine effects [29, 30]. Cell fusion occurs with low frequency but plays an important role in several biological functions, including development, physiology, and disease pathology, and it is classified into two types: homotypic and heterotypic cell fusion [16]. Homotypic fusion occurs between cells of the same lineage, whereas heterotypic fusion occurs between cells of different lineages [31]. Stem cell fusion constitutes heterotypic fusion, and it produces stem cells that have the mature phenotypes of existing cells and are embedded with host cells to enhance cellular function [32–35]. The plasticity characteristics and multilineage differentiation capability of MSCs led to their early use in clinical applications [29]. MSCs differentiate into a diverse range of cell types including osteocytes [36], chondrocytes, cardiomyocytes, hepatocytes, and neuronal lineage cells [37], as shown by evidence from* in vitro* and* in vivo* experiments. Currently, the paracrine and endocrine functions of MSCs are thought to occur through the secretion of various cytokines and growth factors, including vascular endothelial growth factor (VEGF), fibroblast growth factor-2 (FGF-2), insulin growth factor-1 (IGF-1), hepatocyte growth factor (HGF), transforming growth factor- (TGF-) *β*1, prostaglandin E2, and bone morphogenic protein-2 (BMP-2) [38–43]. These factors have beneficial effects on the engraftment of MSCs and host cells through a wide range of biological functions, including immunomodulation [44, 45], proangiogenic [46, 47], antiapoptotic [48, 49], and antioxidative effect [50, 51], and the activation of quiescent progenitor stem cells such that they differentiate and proliferate [52–54]. Additionally, MSC-derived microvesicles, which contain mRNAs, microRNAs, and proteins, exhibit similar biological functions to those described above, and these microvesicles are involved in cell-to-cell communications [16, 55–57]. Thus, microvesicles released from MSCs may also be worth exploiting in stem cell-based therapy. Regarding the secretive paracrine activities of MSCs, there can be a skepticism that questions grounds for using whole cells if the therapeutic potential of MSCs is mainly due to their secretome including microvesicles. In fact, it is highly plausible that the potential of MSCs in regenerating damaged tissues or organs stems from their paracrine activities [58, 59] and that the secretome of MSCs may be proven to be as effective as the whole cell injection in terms of therapeutic effect. Nevertheless, unless delivery of the whole cells to the damaged tissue causes significant adverse effects such as teratoma formation, we believe that using whole cells for therapeutic purposes will retain its significance by providing transient physical reinforcement to the structural integrity of the damaged tissue. For example, MSCs delivered to the coronary artery ligated myocardium with or without hydrogel attenuated ventricular remodeling [60]. In that particular study, hydrogel injection without MSCs also improved cardiac function, and this may suggest that physical reinforcement of the damaged tissue integrity even without paracrine effect of MSCs can accelerate the regeneration process. Therefore, until it is proven that the whole cell injection is absolutely unnecessary to maximize the efficacy of MSC-based cell therapy, the effort to find means to improve MSC adhesion to the host tissue can be justified. In the following sections, we discuss why the transplanted MSCs show low cell viability.

## 3. Causes of MSC Death at the Transplant Site

Transplanted MSCs are confronted with cell death within a few days after transplantation due to a combination of harsh environmental conditions, anoikis, and inflammation [61]. As MSCs have a fibroblastic morphology and attach themselves to the culture plate [62], the first stress occurs when the MSCs are detached from the culture dish in order to prepare for the engraftment process; this induces a decrease in cell viability. Once MSCs are injected into damaged tissues or organs, they encounter a harsh environment (e.g., nutrient and oxygen deprivation) coupled with death signals due to the inadequate tensegrity structure between the cells and matrix. As mentioned above, the lack of matrix support and adhesion to ECM are called anoikis, which promotes apoptotic signaling. Damaged tissues or organs are a result of the pathophysiology of many diseases and lead to oxidative stress, which results from an imbalance between the generation of reactive oxygen species (ROS) and antioxidant mechanisms [63]. This severe condition has a negative effect on engrafted MSCs in the injured area, resulting in increased anoikis [64, 65]. Oxidative stress is also triggered by the inflammatory response, which is essential for promoting angiogenesis and the recruitment of progenitor cells; however, chronic inflammation inhibits the recruitment and survival of progenitor and/or implanted MSCs [66, 67]. Inflammatory cells, such as neutrophils, monocytes, and macrophages, which are recruited by chemokines or cytokines, generate ROS, thereby inducing apoptosis and inactivating the cytoprotective production of nitric oxide (NO) [25, 65].

In the last few years,* ex vivo* manipulation of MSCs has been used to overcome several of the abovementioned limitations, and genetic modifications could enhance the survival, proliferative capacity, and direct differentiation of transplanted MSCs. In addition, pretreatment with bioactive molecules or preconditioning can induce greater paracrine molecules secretion by MSCs, which may result in further therapeutic effects. These approaches are discussed below.

## 4. Improving the Therapeutic Potential of Transplanted MSCs

There are various strategies for strengthening the therapeutic potential of transplanted MSC in order to overcome the low cell survival rates. These approaches involve a variety of treatments including pretreatment with growth factors or cytokines, preconditioning with hypoxia, and the use of genetic modification to overexpress antideath or adhesion signals (Table 1) [25].

### 4.1. Pretreatment with Bioactive Factors and Preconditioning

The biological functions of growth and differentiation factors have recently been studied in an effort to improve the efficacy of implanted MSCs [68]. For example, mouse MSCs with pretreatment IGF-1 exhibited elevated connexin-43, which has both antiapoptotic and cellular proliferation and differentiation functions via gap-junctional intercellular communication, leading to cell reprogramming (prosurvival signaling and cardiomyogenic differentiation) [69]. Similarly, preconditioning with stromal cell-derived factor-1 (SDF-1 or CXCL12), a member of the chemokine CXC subfamily, also suppresses apoptosis, enhances the survival, proliferation, and engraftment of rat MSCs, and improves myocardial function [70]. Heat-shock proteins (Hsps) are molecular chaperones that are involved in the cellular stress response. Our studies demonstrated that rat MSCs transfected with Hsp-70 using the Hph-1 protein transduction domain (PTD) exhibited a protective effect against hypoxia-induced apoptosis and myocardial fibrosis and improved left ventricular function compared with naive MSCs [71].

The hypoxia preconditioning stimulates MSCs, and this effect is considered in the context of comparing normoxia and hypoxia-exposed MSCs* in vitro* or* in vivo* in animal disease models [25, 72]. When mouse MSCs are exposed to hypoxia* in vitro* before transplantation to an infarcted heart, prosurvival and proangiogenic proteins are upregulated, and the implantation of hypoxia-exposed MSCs incudes an increase in angiogenesis and functional recovery [73]. Interestingly, hypoxia preconditioning stimulates the expression and/or secretion of paracrine molecules from MSCs; these molecules include prosurvival and proangiogenic factors, and their expression can result in enhanced cell viability and ability to regenerate [68, 74, 75]. When conditioned medium from hypoxic cultured human MSCs was injected into a rat model of traumatic brain injury, the brain damaged volume and apoptosis were decreased, and motor and cognitive function and neurogenesis were increased [76].

Peng et al. suggested that preconditioning with platelet-rich clot releasate (PRCR), a blood bourn cytoprotective agent with potential for tissue regeneration, can enhance rat MSC survival and proliferation and promote chondrogenic [77] or osteogenic [78] differentiation through the PDGFR-*α*/PI3K/AKT/NF*κ*B signaling pathway [79].

Recent studies have shown that bioactive small molecules and microRNAs are also associated with enhanced MSC adhesion [80, 81]. In our study, the treatment of rat MSCs with a protein kinase C (PKC) activator, phorbol 12-myristate 13-acetate (PMA), increased cell adhesion and significantly decreased detachment, whereas treatment with the PKC inhibitor rottlerin induced slightly diminished cell adhesion. Moreover, following injection into injured rat hearts, the PMA-treated MSCs were retained at a significantly higher rate, and the infarct size, fibrosis area, and number of apoptotic cells were decreased, and cardiac function was improved [80]. In addition, Chinese miniswine MSCs treated with atorvastatin exhibited improved postimplantation survival and enhanced cardiac function via eNOS/NO system [82], and melatonin-pretreated rat MSCs exhibited increased posttransplantation survival, reduced brain infarction, and improved neurobehaviors by activating the ERK signaling pathway in rat cerebral ischemia [83]. Based on a microRNA array, Yu et al. demonstrated that microRNA-125b plays a key role in cell-matrix adhesion and in protecting human MSCs from anoikis by increasing ERK phosphorylation and suppressing p53 expression [81]. Additionally, microRNA-1 transfected mouse MSCs demonstrated enhanced cell survival and cardiomyocyte differentiation, resulting in the recovery of cardiac function after myocardial infarction [84].

In the following section, we will discuss the genetic modification to enhance survival and adhesion of transplanted MSCs.

### 4.2. Genetic Modification to Enhance Antideath Signals

To enhance antideath signaling in MSCs, many studies have focused on specific molecular pathways related to apoptotic and antiapoptotic proteins or the caspase cascade. Toll-like receptor 4 (TLR4), a G protein-coupled receptor, activates proapoptotic signaling under hypoxic conditions, whereas the activation of the PI3K, AKT, and ERK signaling pathway plays a role in improving cell survival. TLR4 knockout murine MSCs show improved survival in spite of hypoxic injury and increased AKT activation [85]. Indeed, TLR4 knockout MSCs produced higher levels of VEGF, HGF, and IGF-1 and recovered myocardial function in rat heart ischemia/reperfusion model through the activation of STAT3 [86]. Mangi and colleagues found that the overexpression of AKT by* ex vivo* retroviral transduction in rat MSCs led to resistance to apoptosis both* in vitro* and* in vivo* and a dramatic improvement in cardiac function in a rodent myocardial infarction model [87].

Hsp-27 is known to have cytoprotective, antioxidant, and anti-inflammatory effects. MSCs transduced with Hsp-27 using a lentiviral vector showed enhanced posttransplantation survival due to the overexpression of prosurvival genes and subsequent caspase cascade inactivation in a rat model of myocardial infarction [88]. Also, Hsp-20 is upregulated by oxidative stress in rat MSCs; thus, MSCs genetically engineered to express Hsp-20 exhibited outcomes similar to those of Hsp-27 modified MSCs due to the activation of AKT and secretion of growth factors (VEGF, FGF-2, and IGF-1) [89].

Rat MSCs genetically modified by the jetPEI-mediated transfection (nonviral vector, DNA transfection reagent) of the antiapoptotic gene Bcl-2 exhibit decreased cell death and upregulation of the angiogenic cytokine VEGF. Upon transplantation Bcl-2-transfected MSCs showed increased cellular survival and functional recovery of infarcted heart [90]. Fan et al. demonstrated that rat MSCs modified by the lentivirus-driven expression of survivin, a member of the inhibitor of apoptosis protein family (IAP), exhibited an enhanced survival rate after transplantation and showed better therapeutic effects for stroke [91] and myocardial infarction [92].

Chemokines and their receptors play an important role in stem cell homing, chemotaxis, and adhesion. To enhance these effects, mouse MSCs transduced with chemokine (c-c motif) receptor 1 (CCR1) or chemokine (c-x-c motif) receptor 2 (CXCR2) exhibited improved engraftment and survival in injured myocardium [93].

### 4.3. Genetic Modification to Enhance Cell Adhesion

**Figure 1:**
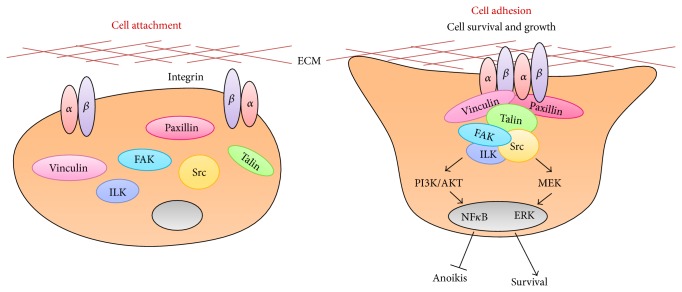
Cell adhesion to the extracellular matrix is mainly mediated by integrins. Integrins are associated with cell-to-cell and cell-to-extracellular matrix (ECM) adhesion events via ECM binding and/or cell adhesion molecules. Loss of integrin attachment induces anoikis, which leads to cell death. The focal adhesion complex, which includes focal adhesion kinase (FAK), Src, talin, vinculin, paxillin, and integrin-linked kinase (ILK), promotes the strong adhesion and cell survival/growth of the transplanted stem cells by inhibiting anoikis signaling.

As discussed earlier, the adhesion of the transplanted MSCs is potentially a major contributor to cell engraftment and tissue/organ regeneration, and the detachment of transplanted MSCs results in anoikis (Figure 1). Integrins are associated with ECM interactions and connexins (Cxs) are associated with cell-cell and cell-matrix interactions, which are thought to regulate stem cell survival and proliferation [94]. To avoid anoikis and improve the survival of implanted MSCs, the regulation of molecules involving cell attachment may be considered an important area of study.

Using microarray and proteomic screening, Copland et al. identified plasminogen activator inhibitor 1 (PAI-1) as upregulated in mouse and human MSCs under hypoxic conditions. The MSCs isolated from PAI-1 knockout mice showed more survival and adhesiveness than wild-type MSCs after transplantation on Matrigel. These findings show that PAI-1 negatively regulates transplanted MSC survival and adhesiveness via promoting anoikis [95].

In a previous study, we genetically engineered rat MSCs to overexpress tissue transglutaminase (tTG) using Lipofectamine. The tTG acts as a coreceptor for fibronectin (Fn) in cell adhesion associated with integrins [96, 97]. The overexpression of tTG in rat MSCs enhances cell attachment, spreading, and migration through the formation of focal adhesion complexes and the increased phosphorylation of focal adhesion-related kinases including focal adhesion kinase (FAK), Src, and PI3K. Furthermore, the implantation of tTG-transfected MSCs in rat infarcted myocardium restored cardiac functions [97]. Additionally, the activation of FAK is regulated by integrins and can suppress anoikis. Cell-cell and cell-matrix adhesion are interrupted by ROS generation, which is associated with a decrease in the level of focal adhesion-related molecules (such as phospho-FAK, Src, and *α*V*β*1 integrin) in the transplanted MSCs in hostile microenvironments [64, 72].

Another study has also demonstrated that integrin-linked kinase (ILK) is essential to strengthen cell adhesion to the ischemic myocardium in hypoxic rat MSCs. ILK is a Ser/Thr kinase that interacts with the cytoplasmic domain of *β*1 integrin and plays a crucial role in integrin-mediated cell adhesion and signaling [25]. Our data suggested that the ILK transfection of MSCs using a lentiviral vector can enhance cell survival and adhesion and prevent anoikis [98, 99]. We also observed that ILK increases the phosphorylation of ERK and AKT, which are involved in the regulation of adhesion-mediated cell survival signaling in hypoxic MSCs, and also increases the Bcl-2/Bax ratio and inhibits caspase-3 activation. The transplantation of ILK-transduced MSCs into rat infarcted myocardium resulted in reductions in infarct size, apoptotic signaling, and fibrosis and in improved microvessel density [72, 99].

Together, these studies demonstrated that* ex vivo* genetic modification of MSCs provides a benefit by enhancing cell survival and adhesion, as well as therapeutic potency, in cells transplanted to sites of injury. Furthermore, enhancing adhesion and migration of MSCs by deliberate modifications might benefit the MSC-based systemic therapies that exploit MSC's tropisms for tumor and inflammation sites [100, 101], by guiding proper migration and permanent anchorage to target tissue. For example, there has been attempt to use MSCs as drug delivery vehicle for treating cancer [102, 103]. In such application, adequate modification of MSCs can improve migration and integration of MSCs in the targeted tissue, increasing efficacy of the therapy. Taken together, enhancing cell adhesion may have considerable positive effects on the efficacy of MSC-based therapies.

## 5. Conclusions and Future Considerations

The abilities of MSCs to self-renew and differentiate into various cell types have made these cells a major resource for stem cell-based therapies. The putative mechanisms by which transplanted MSCs exert regenerative effects in injured tissues include differentiation, cell fusion, and paracrine effects, such as immunosuppressive and antiapoptotic effects and the stimulation of local progenitor stem cells. Many studies have demonstrated that MSC therapy is safe and effective in preclinical and clinical trials. However, a major obstacle in MSC therapy is the low survival rate after transplantation due to cell death via anoikis. To improve the survival and cell adhesion of the transplanted MSCs, various strategies have been investigated, including pretreatment with growth factors or cytokines, hypoxic preconditioning, and genetic modifications to induce the overexpression of antiapoptotic signaling or adhesion molecules (Figure 1; Table 1). These strategies have enhanced the therapeutic efficacy of MSCs in repairing damaged tissues. Prerequisites for the broad application of MSCs include determining the exact mechanisms underlying their regenerative actions, identifying the involved molecules, and addressing unsolved issues relating to their safety, administration route, and the appropriate amount of MSCs to inject, as well as practical considerations such as large-scale culture conditions, storage, and distribution strategies. Therefore, a better understanding of these risk factors may contribute to the improvement of stem cell-based therapies. Additionally, optimized, standardized, and controlled methods of exogenous MSC delivery are being developed; the relevant preclinical and clinical studies will contribute to a fuller understanding of the effects of microenvironment on stem cell activity and repair mechanisms, which will in turn facilitate the success of regenerative medicines using MSCs.

## Figures and Tables

**Table 1:** Potential strategies for enhancing MSC survival and adhesion.

Types	Related factors	Function	Reference
*Ex vivo genetic modification *			
Antiapoptosis	TLR4	Improved survival of TLR4 knockout murine MSCs	[85, 86]
	AKT	Reduced apoptosis of rat MSCs	[87]
	Hsp-20/Hsp-27	Enhanced survival of rat MSCs	[88, 89]
	Bcl-2	Inhibited cell death of rat MSCs	[90]
	Survivin	Enhanced survival of rat MSCs	[91, 92]
	CCR1/CXCR2	Improved survival of mouse MSCs	[94]
Cell adhesion	PAI-1	Enhanced survival and adhesion of PAI-1 knockout mouse MSCs	[95]
	tTG	Enhanced cell attachment of rat MSCs	[96, 97]
	ILK	Enhanced cell survival and adhesion of rat MSCs	[98, 99]
Pretreatment	IGF-1	Elevated connexin-43 and enhanced prosurvival signals and cardiomyogenic differentiation of mouse MSCs	[69]
	SDF-1	Suppressed apoptosis, enhanced survival, proliferation, and engraftment of rat MSCs	[70]
	Hsp-70	Protected against hypoxia-induced apoptosis of rat MSCs	[71]
	PRCR	Enhanced survival, proliferation, and differentiation of rat MSCs	[77–79]
	PMA	Increased adhesion of rat MSCs	[80]
	Atorvastatin	Improved survival of swine MSCs	[82]
	Melatonin	Increased survival of rat MSCs	[83]
	MicroRNA-1	Enhanced survival and cardiomyocyte differentiation of mouse MSCs	[84]
	MicroRNA-125b	Protected against anoikis in human MSCs	[81]
Preconditioning	Hypoxia	Expressed and secreted prosurvival and proangiogenic factors from MSCs	[25, 68, 72–76]

TLR4: Toll-like receptor 4; Hsp: heat-shock protein; CCR1: chemokine (c-c motif) receptor 1; CXCR2: chemokine (c-x-c motif) receptor 2; PAI-1: plasminogen activator inhibitor 1; tTG: tissue transglutaminase; ILK: integrin-linked kinase; IGF-1: insulin growth factor-1; SDF-1: stromal cell-derived factor-1; PRCR: platelet-rich clot releasate; PMA: phorbol 12-myristate 13-acetate; MSCs: mesenchymal stem cells.
